# Acute Hemorrhagic Leukoencephalitis: A Case and Systematic Review of the Literature

**DOI:** 10.3389/fneur.2020.00899

**Published:** 2020-08-20

**Authors:** Pascale Grzonka, Marleen C. Scholz, Gian Marco De Marchis, Kai Tisljar, Stephan Rüegg, Stephan Marsch, Joachim Fladt, Raoul Sutter

**Affiliations:** ^1^Intensive Care Units, University Hospital Basel, Basel, Switzerland; ^2^Department of Neurology, University Hospital Basel, Basel, Switzerland; ^3^Medical Faculty, University of Basel, Basel, Switzerland

**Keywords:** leukoencephalitis, immunosuppressive therapy, outcome, mortality, parainfectious disease

## Abstract

**Objectives:** To present a patient with acute hemorrhagic leukoencephalitis (AHLE) and a systematic review of the literature analyzing diagnostic procedures, treatment, and outcomes of AHLE.

**Methods:** PubMed and Cochrane databases were screened. Papers published since 01/01/2000 describing adult patients are reported according to the PRISMA-guidelines.

**Results:** A 59-year old male with rapidly developing coma and cerebral biopsy changes compatible with AHLE is presented followed by 43 case reports from the literature including males in 67% and a mean age of 38 years. Mortality was 47%. Infectious pathogens were reported in 35%, preexisting autoimmune diseases were identified in 12%. Neuroimaging revealed uni- or bihemispheric lesions in 65% and isolated lesions of the cerebellum, pons, medulla oblongata or the spinal cord without concomitant hemispheric involvement in 16%. Analysis of the cerebrospinal fluid showed an increased protein level in 87%, elevated white blood cells in 65%, and erythrocytes in 39%. Histology (reported in 58%) supported the diagnosis of AHLE in all cases. Glucocorticoids were used most commonly (97%), followed by plasmapheresis (26%), and intravenous immunoglobulins (12%), without a clear temporal relationship between treatment and the patients' clinical course.

**Conclusions:** Although mortality was lower than previously reported, AHLE remains a life-threatening neurologic emergency with high mortality. Diagnosis is challenging as the level of evidence regarding the diagnostic yield of clinical, neuroimaging and laboratory characteristics remains low. Hence, clinicians are urged to heighten their awareness and to prompt cerebral biopsies in the context of rapidly progressive neurologic decline of unknown origin with the concurrence of the compiled characteristics. Future studies need to focus on treatment characteristics and their effects on course and outcome.

## Introduction

Acute hemorrhagic leukoencephalitis (AHLE) is an inflammatory disease of the brain, most often affecting the cerebrum, less commonly the cerebellum, the brain stem, or the spinal cord. Weston Hurst was the first to describe this syndrome in 1941, reporting two adults who developed severe and rapidly progressive encephalopathy due to hemorrhagic lesions of the white matter, histologically characterized by perivascular polymorphonuclear infiltrates, small vessel necrosis and demyelination.

AHLE is commonly considered to be a variant of acute disseminated encephalomyelitis (ADEM) (1, 2). While the latter is mainly seen in children, the former is more common in adults. Due to the rarity of the disease and the complex diagnostic workup, AHLE is likely to be underrecognized, and underreported.

The etiology of AHLE is unknown. The initial emergence of flu-like symptoms, however, supports the hypothesis of an autoimmune process on the basis of molecular mimicry promoted by mostly viral or bacterial pathogens. In keeping with this, immunosuppressive therapy, mainly with glucocorticoids, is the mainstay of treatment. Based on case reports and small case series, mortality is reported to be as high as 70% (3, 4).

In order to heighten awareness of AHLE and its clinical context, we present an adult patient with typical features of AHLE and a systematic review of the literature aiming to analyze the diagnostic procedures, treatment options, and outcomes of AHLE.

## Methods

The digital databases PubMed and Cochrane were screened by two reviewers using predefined search terms in the advanced search mode. The term “acute hemorrhagic leukoencephalitis” was applied as a MESH term as well as in “title/abstract.” We included all papers meeting each of the following criteria: (1) the papers had to be published after 01/01/2000, (2) the papers had to be describing adult patients (age ≥ 18 years), (3) the publication had to be written in English, and (4) the design had to be either case reports, case series or cohort studies. The results are reported according to the PRISMA-guidelines (http://www.prisma-statement.org).

**Figure 1:**
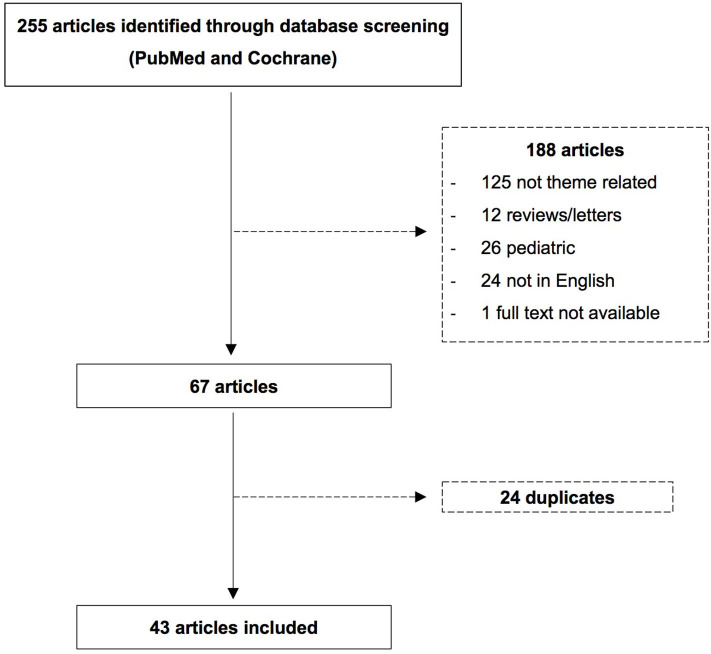
Flow chart.

Details regarding search terms as well as in- and exclusion processes are outlined in Figure 1. Data regarding demographics, clinical and neuroradiologic characteristics were extracted.

## Case Report

**Figure 2:**
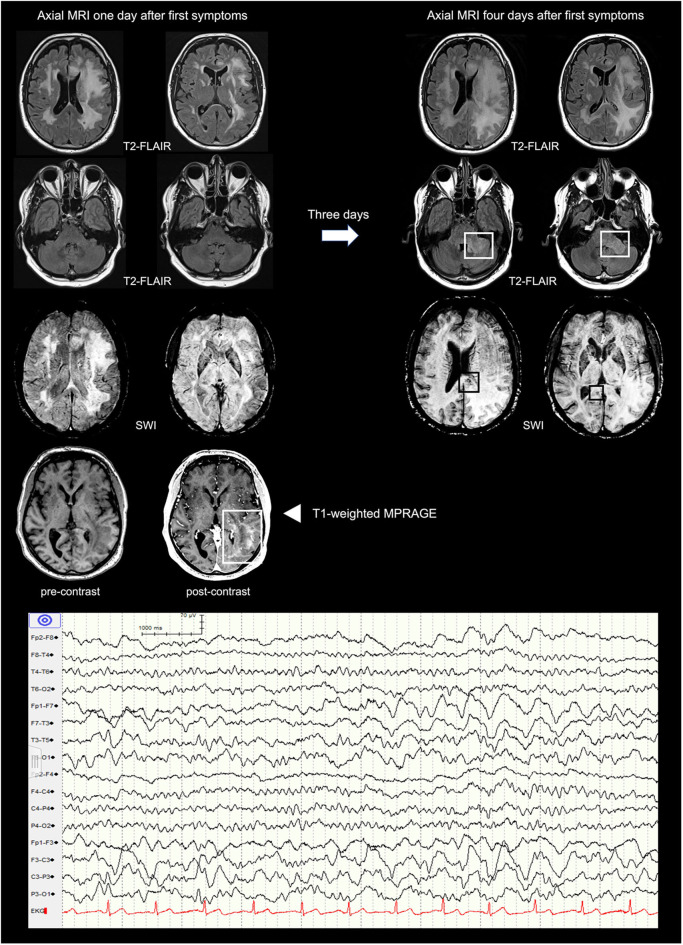
Cerebral MRI presenting the temporal evolution within 3 days (from left to right) and EEG excerpt. First row: axial T2-weighted FLAIR images showing increasing bilateral confluent widespread hyperintensities of the supratentorial white matter predominantly on the left. Second row: axial T2-weighted FLAIR images revealing new hyperintensities of the left cerebellar peduncle. Third row: axial SWI demonstrating subtle and small susceptibility artifacts in the splenium of the corpus callosum. Fourth row: axial pre- and post-contrast T1-weighted MPRAGE showing enhancement of the left parieto-occipital region. FLAIR, Fluid-Attenuated Inversion Recovery; SWI, Susceptibility Weighted Imaging; MPRAGE, Magnetization-Prepared Rapid Acquisition with Gradient Echo.

A 59-year old caucasian male with a history of recurrent pulmonary embolisms, obstructive sleep apnea syndrome, and psoriasis, as well as an unspecific infection of the respiratory tract infection 2 weeks prior to presentation was referred to our tertiary academic medical care center with suspected wake-up stroke. Clinical examination revealed new onset aphasia, right sided central facial and brachial paralysis. No neck stiffness was noted and inflammatory parameters in the peripheral blood were only mildly elevated [white blood cell count (WBC) 10.9 × 10^9^ per liter, C-reactive protein 13 milligram per liter]. Repeat cerebral CT scans were unremarkable, whereas analysis of the cerebrospinal fluid (CSF) revealed a high leukocyte count (1,074 per microliter), an elevated lactate concentration (4.6 mmol per liter), an increased protein level (2.3 gram per liter) and ferritin (85 microgram per liter). Empiric antibiotic and antiviral therapy with ceftriaxone, amoxicillin and aciclovir was initiated. An extensive microbiologic and rheumatologic workup was negative. Cerebral MRI 1 day after first symptoms mainly demonstrated left-hemispheric hyperintense lesions of the white matter (Figure 2; left part of the first row) and an enhancement of the left parieto-occipital regions (Figure 2; fourth row).

After a temporary slight improvement, the patient deteriorated rapidly with symptoms evolving to mutism, right sided hemiplegia and finally deep coma with a Glasgow coma scale (GCS) of 3 within 4 days. Repeat cerebral MRI 3 days later revealed a progression of the white matter lesions now involving the cerebellum, the ponto-medullar region, and both hemispheres with cerebral edema resulting in a mild midline shift (Figure 2; right part of the first row). In addition, susceptibility artifacts in the splenium of the corpus callosum and the pedunculus cerebelli were consistent with multiple but subtle micro bleeds. The electroencephalogram (EEG) showed intermittent epileptiform activity in both hemispheres with left fronto-temporal predominance (Figure 2; lower part).

**Figure 3:**
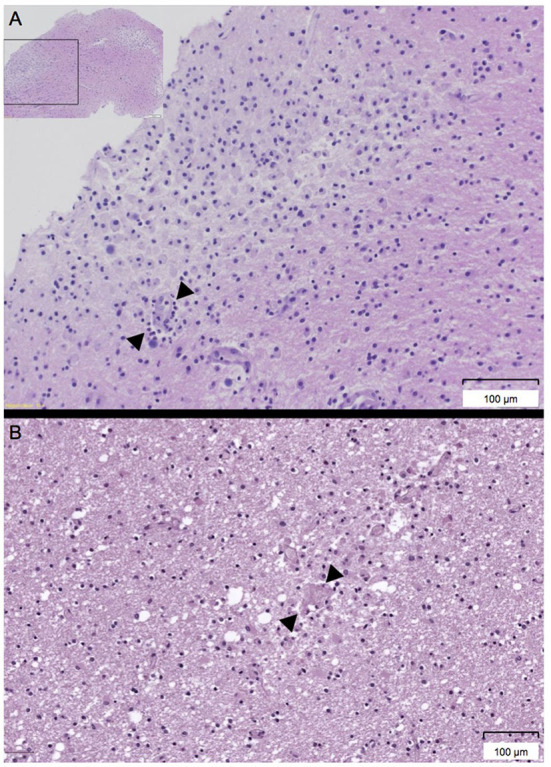
**(A,B)** Histologic workup of the biopsy of the left frontal lobe and the cerebral autopsy. **(A)** Histology of the biopsy of the left frontal lobe showing perivascular infiltrates (arrow) of neutrophils, eosinophils, and macrophages (Hemalaun Eosin [HE] stain). **(B)** Histology of the cerebral autopsy revealing diffuse generalized inflammation and acute hemorrhages (arrow) (Hemalaun Eosin [HE] stain).

A biopsy of the left frontal lobe showed infiltrates with neutrophilic and eosinophilic granulocytes as well as macrophages, acute focal hemorrhages, and vasculitis-like vessel lesions compatible with AHLE (Figure 3A). Despite intensive immunosuppressive therapy including intravenously administered immunoglobulins (IVIG; 0.4 g per kg body weight for 5 days), high dose glucocorticoids (methylprednisolone 2 gram per day for 3 days followed by tapering) and cyclophosphamide (15 milligram per kilogram body weight), the patient showed no improvement and remained deeply comatose with a loss of protective reflexes. Best supportive care was started 13 days after admission and the patient died shortly after.

Autopsy revealed disseminated, mainly perivascular demyelination with focal hemorrhages (Figure 3B) located in both hemispheres, the corpus callosum and the pons with cortical sparing, compatible with AHLE.

## Results From Systematic Review

Of the 255 articles screened from the digital databases PubMed and Cochrane, 43 met our inclusion criteria, each contributing one patient. Males were more commonly affected than females (67 vs. 33%) and patients' mean age was 38 years. Detailed data extracted from these reports are outlined in Table 1.

**Table 1:** Clinical and neuroradiologic characteristics of adult patients with AHLE.

**Ref. No. Year of publication**	**Age**	**Sex**	**Infectious and non-infectious associated conditions**	**Neuroimaging findings associated with AHLE**	**Pathological CSF findings**	**Immunosuppressive treatment**	**Outcome**
(5), 2000	34	Male	Not reported	Frontal, temporal and parietal lobe, right basal ganglia	WBC 64 (mainly mononuclear), RBC 124, protein 1.25	Dexamethasone 24 mg/d	full recovery
(6), 2001	41	Female	Sepsis with *Staph. epidermidis* Crohn's disease	Diffuse swelling in the posterior fossa, no focal lesion (CT)	RBC 4880, protein 1.09	Not reported	death (AHLE diagnosed post-mortem)
(7), 2001	44	Female	Upper respiratory tract infection 1 week prior	Bilateral hemispheres	WBC 103 (mainly polynuclear), protein 0.98	Dexamethasone 15 mg/d, later Methylprednisolone 1 g/d, EVD	full recovery
(8), 2002	28	Male	*Mycoplasma pneumoniae*	Right hemisphere	Not reported	Decompressive craniectomy, glucocorticoids	survival (minor hemiparesis left, homonymous hemianopia to the left, neglect)
(9), 2003	19	Male	Upper respiratory symptoms 2 weeks prior	Bilateral posterior hemispheres, splenium of corpus callosum	Not reported	Dexamethasone	death
(10), 2004	57	Female	Flu-like symptoms 7 days prior	Frontal and temporal lobes	WBC 58 (mainly mononuclear), protein 2.85	Methylprednisolone 1 g/d	survival (global aphasia, tetraparesis)
(11), 2004	28	Male	*EBV* (*EBV* DNA in CSF and brain biopsy)	Bilateral temporal lobes, thalamus	WBC 24 (mainly mononuclear), protein 0.65	Dexamethasone 16 mg/d	death
(12), 2005	43	Male	Upper respiratory tract infection 2 weeks prior	Frontal and temporal lobes, generalized edema	RBC 37, protein 0.75	Dexamethasone, EVD	survival (mild left hemiparesis)
(13), 2005	42	Female	Not reported	Bilateral frontal lobes, corpus callosum, left thalamus, capsula interna	WBC 2100 (mainly polymorphonuclear), protein 1.76	Prednisolone	survival (with residual neurological deficits)
(14), 2006	22	Female	Not reported	Bilateral hemispheres	Not reported	Methylprednisolone 1 g/d	full recovery
(15), 2007	31	Male	*Mycoplasma pneumoniae*	Not reported	Elevated myelin basic protein, cell count, and protein not reported	Dexamethasone, plasmapheresis, EVD, decompressive craniectomy	survival (slight upper extremity tremor)
(16), 2009	30	Male	*Mumps*	Parieto-occipital lobes, thalami, cerebellum, brainstem	Not reported	Methylprednisolone	death
(17), 2009	62	Male	*Mycoplasma* pneumonia 2 weeks prior	Right cortex, left corpus callosum, pons, mesencephalon	Normal	Dexamethasone 32 mg/d, plasmapheresis	survival (left hemiplegia)
(18), 2010	20	Male	*EBV* (PCR brain biopsy)	Right temporal lobe	WBC 14 (mainly mononuclear), protein 1.6	Decompressive craniectomy, EVD	full recovery
(19), 2010	76	Male	*EBV* (PCR CSF) polyarteritis nodosa	Cerebellum	WBC 10 (mononuclear), protein 0.78	Methylprednisolone 500 mg/d	survival
(20), 2010	40	Male	*Influenza H1N1*	Bilateral hemispheres	Protein 2.4, RBC 157	Not reported	survival (severely disabled)
(21), 2010	21	Male	Not reported	Pons	WBC 25, protein 0.7	Methylprednisolone 1 g/d, plasmapheresis	death
(22), 2011	25	Male	Not reported	Bilateral hemispheres, brainstem, and cerebellum	Protein 0.53	Methylprednisolone	survival with mild left hemiparesis
(23), 2011	56	Female	*Influenza H1N1*	Bilateral cerebral hemispheres, right striatum	RBC 90, WBC 8 (mononuclear), protein 0.51	Methylprednisolone 500 mg/d	survival (mild action tremor)
(24), 2011	70	Male	Non-specific abdominal pain with fever 1 week prior	Both cerebral hemispheres, brainstem, medulla oblongata	WBC max. 267 (mainly polymorphonuclear), RBC max. 400, protein max 72	Methylprednisolone 1 g/d, IVIG	death
(25), 2011	37	Male	Not reported	Cerebellum, medulla oblongata	WBC max. 700 (mainly mononuclear), RBC max. 100, protein max. 1.56	Glucocorticoids	survival (right-sided weakness, ataxia and dysarthria)
(26), 2011	23	Male	Not reported	Pons, medulla oblongata, proximal spinal cord	WBC 20 (mononuclear), protein 0.69	Methylprednisolone 1 g/d, followed by prednisolone 40 mg/d	death
(27), 2012	51	Male	Gastroenteritis 2 weeks prior	Bilateral hemispheres	Not reported	Dexamethasone	death
(28), 2013	27	Male	*Influenza H1N1*	Not performed	Not reported	Not reported	death (AHLE diagnosed post-mortem)
(29), 2013	22	Male	*Plasmodium vivax* (blood smear)	Bilateral occipital and parietal lobes	WBC 1400 (mainly polymorphonuclear), protein 1.2	Prednisolone	death
(30), 2014	75	Male	Rheumatoid arthritis, hypothyroidism	Medulla oblongata, pons, cerebellum	WBC 90 (mainly polymorphonuclear), RBC 101, protein 1.84	Not reported	death
(31), 2014	39	Male	Flu-like symptoms 3 days prior	Normal (CT)	WBC 365 (mainly mononuclear), RBC elevated, protein 4.66	Not reported	death (AHLE diagnosed post-mortem)
(3), 2014	24	Female	Autoimmune myopathy	Right frontal lobe	Not reported	Decompressive craniectomy dexamethasone, plasmapheresis	full recovery
(32), 2015	48	Male	Pneumonia, viral myocarditis	Not performed	Not performed	Not reported	death (found dead)
(1), 2015	21	Female	Multiple sclerosis	Bilateral basal ganglia	Protein 1.2	Prednisolone	survival (severely disabled)
(33), 2016	34	Female	Upper respiratory symptoms 6 weeks prior possibly due to *Coxsackie B6* virus	Pons, right cerebellum, frontal lobe, bilateral hippocampi	Protein 0.48	Methylprednisolone, IVIG	death
(34), 2016	27	Male	*Mycoplasma pneumoniae* (PCR of brain biopsy)	Right frontal and parietal lobe	Not reported	Dexamethasone 12 mg/d, decompressive craniectomy, partial frontal lobectomy, EVD	death
(35), 2016	44	Male	Snake bite (Russell's viper)	Frontal, parietal and temporal lobes	Not reported	Not reported	full recovery
(36), 2016	25	Female	Upper respiratory tract infection 2 weeks prior	Brainstem, corpus callosum	Protein 0.65	Methylprednisolone 1 g/d, plasmapheresis, mannitol	survival (severely disabled)
(37), 2016	33	Female	*Influenza* vaccination 2 weeks prior	Spinal cord (C7–T11)	WBC 55, RBC 2050	Methylprednisolone 1 g/d, IVIG	survival (paraplegia)
(38), 2017	33	Female	None	Right fronto-parietal and temporo-parietal lobes	Normal (after 26 days of treatment)	Dexamethasone 16 mg/d	survival (left-sided apraxia)
(4), 2017	19	Male	Isolated fever for 2 weeks	Left parietal, occipital and frontal regions, corpus callosum, left basal ganglia	WBC 45 (mainly polymorphonuclear), few RBCs, protein 0.51	Methylprednisolone, IVIG, cyclophosphamide, rituximab, plasmapheresis, craniectomy, hypertonic saline	death
(39), 2017	36	Female	Pregnancy, colitis ulcerosa, primary sclerosing cholangitis	Frontal lobes, corpus callosum, basal ganglia	RBC 110, protein 0.52	Methylprednisolone, plasmapheresis	death
(40), 2017	36	Male	Not reported	Not performed	Not reported	None	death (AHLE diagnosed post-mortem)
(41), 2018	70	Male	*Influenza* revaccination 3 days prior	Bilateral hemispheres, corpus callosum, posterior brain stem	WBC 199, (mainly polymorphonuclear), protein 1.74; follow-up CSF acellular, protein 8.53	Methylprednisolone 1 g/d, plasmapheresis	death
(2), 2018	25	Female	Flu-like symptoms 3 weeks prior	Cerebellum	WBC 13 (mononuclear), protein 2.86	Glucocorticoids, plasmapheresis, decompressive craniectomy, VP-shunt	survival (nystagmus, minimal dysarthria)
(42), 2019	63	Male	*HSV* (PCR from CSF)	Bilateral fronto-temporo-parietal hemispheres	WBC 58 (mainly polymorphonuclear), RBC 70, protein 0.7	Dexamethasone 0.15 mg/kg body weight/day, methylprednisolone 1 g/d	survival (severely disabled)
(43), 2019	42	Male	Cough and fever about 1 week prior	Brainstem, especially right pons, right temporo-occipital hemorrhage	Not reported	Not reported	death

*Units: cell count (RBC/WBC), number per microliter; protein, gram per liter*.

*CSF, cerebrospinal fluid; WBC, white blood cells; RBC, red blood cells; IVIG, intravenous immunoglobulins; EVD, external ventricular drainage; VP-shunt, ventriculo-peritoneal shunt; PCR, polymerase chain reaction*.

As in our patient described above who initially suffered from a respiratory tract infection, infectious pathogens in the context of AHLE were reported in the literature in 35%, including *Staphylococcus epidermidis* (6), *Epstein Barr virus* (*EBV*) (11, 18, 19), *Influenza H1N1* (20, 23, 28), *Coxsackie* B6 (33), *Cytomegalovirus* (*CMV*), *Human Herpes virus 6* (*HHV-6*), *Herpes simplex* (*HSV*), (*Varicella zoster* (*VZV*) (42, 44), *Mumps* virus (16), *Mycoplasma pneumoniae* (8, 15, 17, 34), *Plasmodium vivax* (29), and *Mycobacterium tuberculosis* (45).

Symptoms of upper respiratory tract infections without identification of the underlying pathogen were described in 19%. In two patients, AHLE occurred after *influenza* vaccination (37, 41). As in our patient, who suffered from psoriasis, preexisting autoimmune disease is frequently reported in the literature, such as rheumatoid arthritis (30), inflammatory bowel disease (6, 39), primary sclerosing cholangitis (39), multiple sclerosis (1), and polyarteritis nodosa (19) was present in 12%.

Neuroimaging was performed in 91% of all cases. Uni- or bilateral hemispheric lesions were most frequently reported (in 65%), whereas isolated lesions of the cerebellum, the pons, the medulla oblongata, or the spinal cord without concomitant hemispheric involvement were rare (16%).

CSF analysis was reported in 72%. As seen in the CSF of our patient, the most frequent finding reported in the literature was an increased protein level (87%). WBC was elevated in 65% (of which 50% mainly mononuclear and 40% mainly polymorphonuclear), RBC in 39%.

A histologic diagnostic work-up was performed in 58% of patients (biopsy in 26%, autopsy in 35%, both biopsy and autopsy in 1 patient). In all cases providing histologic work-up, the findings supported the diagnosis of AHLE.

Treatment was described in 79%. Glucocorticoids were the most common immunosuppressive therapy applied (97%), followed by plasmapheresis (26%), and intravenous immunoglobulins (12%). In one patient, the use of cyclophosphamide and rituximab was reported. However, a clear temporal relationship between immunosuppressive therapy and the patients' clinical course could not be established in most case reports.

Overall mortality was 46.5%. Fourteen percentage of patients made a full recovery, whereas 39.5% survived with mild to severe neurological impairment.

Table 2 presents a comparison of clinical, neuroradiologic, and laboratory differences between AHLE and ADEM based on the data of our systematic review and recent case reports and reviews regarding ADEM.

**Table 2:** Comparison of main clinical, neuroradiologic, and laboratory characteristics between AHLE and ADEM.

		**ADEM**	**AHLE**
Age		- More common in children	- More common in adults
Epidemiology		- Incidence 0.3–0.6 per 100,000 per year (46) - Male predominance (46) - More common in children and teenagers (33)	- Incidence unknown - Male predominance (see Table 1)
Clinical findings	Symptoms	- Focal neurological symptoms according to the location of lesions - Unspecific encephalopathy (12)	- Focal neurological symptoms according to the location of lesions - Symptoms and signs of elevated intracranial pressure possible (12)
	Clinical course	- Less fulminant, coma unusual (12)	- Fulminant, frequently evolving to coma/death within days (12)
Radiological findings	FLAIR	- Hyperintense lesions of the white matter (12)	- Larger lesions, confluent - Significant edema with space-occupying effect (9, 12)
	SWI/T2*	- no data	- (Petechial) hemorrhages (12)
Laboratory and histological findings	CSF	- Protein increased in 23–62% of pediatric patients (46) - Lymphocytic pleocytosis (12, 25)	- Granulocytic pleocytosis (12) - Elevated protein in 87% (see Table 1) - Normal glucose (12) - Possibly erythrocytes/ferritin elevated
	Peripheral blood	- No leukocytosis (12)	- Neutrophil-predominant leukocytosis (12)
	Histopathology	- Perivascular demyelination with lymphocytic infiltration (9)	- Necrosis of small vessels (12) - Perivascular fibrin exudation (31) - Hemorrhages (“ring-and-ball”) (9) - Infiltration with neutrophils and macrophages (31) - Demyelination in later stages (31)
Treatment options		- Glucocorticoids - IVIG - Plasmapheresis	- Glucocorticoids - IVIG - Plasmapheresis - Cyclophosphamide, rituximab
Prognosis		- Favorable - Complete remission in 60–80% (12) - Mortality 1–3% (46)	- Unfavorable - Complete remission in 14% (see Table 1) - Surviving patients with significant neurological sequelae (12) - Mortality 46.5% (see Table 1)

*ADEM, acute disseminated encephalomyelitis; AHLE, acute hemorrhagic leukoencephalitis; FLAIR, Fluid attenuated inversion recovery; SWI, susceptibility weighted imaging; CSF, cerebrospinal fluid; IVIG, intravenously administered immunoglobulins; numbers in brackets, corresponding references*.

## Discussion

AHLE is a rare disease with a rapidly progressive course and prompt recognition is crucial. However, since no evidence-based diagnostic criteria exist, diagnosis is challenging. The limited number of cases described in the literature, likely reflecting the low incidence and/or underreporting, calls for heightened awareness in the context of patients developing acute cerebral inflammation of unknown origin. Since we could not identify formal studies and our insights are based on case reports only, the level of evidence regarding the clinical, neuroimaging, and laboratory characteristics remains very low. However, as mentioned above, our patient presented many of the typical clinical features of AHLE as described in the literature that may assist in the diagnostic workup as follows:

First, a preceding or concomitant infection is reported in more than 50%, with different viruses being the most commonly reported pathogens, for example EBV, mumps, VZV, HSV, HHV-6, and influenza. In 19% of all patients with AHLE, symptoms of a non-specific upper respiratory tract infection without identification of an underlying pathogen are described, as was the case in our patient.

Second, the fact that the illness emerged in a male adult person is typical. In contrast to ADEM, which is more common in children and teenagers (33), AHLE mainly affects adult patients. Moreover, our literature review shows a male preponderance of 67%.

Third, the clinical course with rapid neurological decline eventually leading to coma and death also suggests AHLE. In the literature, mortality is mentioned to be as high as 70% (3, 4). However, in our review of the literature, we found an overall mortality of 46.5%, which is substantially lower. Moreover, 14% of patients made a full recovery and returned to their premorbid neurological baseline (3, 5, 7, 14, 18, 35), and 11% survived with only minor neurological sequelae (2, 12, 15, 23, 38). The reason for the better outcome in this systematic review compared to previous studies (3, 4) remains unclear. The fact that aggressive immunosuppression was described in a large proportion of cases, however, suggests a treatment-related improvement. Unfortunately, if and to what extent aggressive immunosuppression influences outcome cannot be determined by our data and other factors that may play an important role regarding disease control remain to be uncovered.

The most frequently discussed pathomechanistic hypothesis is an autoimmune process promoted by cross reactivity (i.e., molecular mimicry) between human myelin and viral or bacterial antigens, but the exact mechanisms remain to be elucidated (33).

Diagnosis of AHLE mainly relies on neuroimaging, cerebrospinal fluid (CSF) analysis and histopathology. Due to the lack of formal studies, guidelines defining diagnostic algorithms are lacking, and cannot be drawn from current data. Furthermore, most clinical scenarios described in the literature encompass symptoms and signs that may prompt the clinician to suspect ADEM. While single clinical characteristics do not reliably differ between AHLE and ADEM, the concurrence of multiple symptoms, and signs may facilitate the distinction between these two entities. In this context, Table 2 presents a compilation of different symptoms and diagnostic findings that are most discriminative between AHLE and ADEM. However, as the syndromatic appearance and clinical course of AHLE and ADEM are often indistinguishable, treatment options are equal, and the body of evidence regarding the latter is limited for both entities, the need for a reliable differentiation seems questionable.

Brain MRI is crucial and typically reveals confluent white matter lesions with significant edema, space-occupying effects, and petechial hemorrhages (12, 38). The location of the lesions seems highly variable. Uni- or bilateral hemispheric involvement is seen in nearly two third of patients, but other distributive patterns have been described. The most useful aspect on cerebral MRI in differentiating AHLE from ADEM is, however, the presence of intraparenchymal hemorrhages.

While infectious encephalitides are more frequent and may represent an important differential diagnosis to AHLE, their diagnosis is usually less challenging, as multiplex PCR assays and whole genome sequencing approaches in the cerebrospinal fluid allow rapid detection of several infectious pathogens including HSV type 1, the most commonly identified cause of sporadic encephalitis worldwide that mostly affects the temporal lobe and limbic region (47). A study of adult immune competent patients with encephalitis who had temporal lobe abnormalities found that bilateral temporal lobe involvement was associated with lower odds of HSV encephalitis compared to all other etiologies (48). Moreover, patients with Herpes encephalitis were more likely to be older and white, and to present acutely and with fever, seizures, and upper respiratory symptoms. In addition to the bilateral temporal lobe involvement, lesions outside the temporal lobe or limbic region suggested alternative diagnoses and thus may also help to differentiate Herpes encephalitis from AHLE. Early recognition is crucial, as treatment of the former entity is effective with the intravenous administration of aciclovir, as well as screening for and treatment of limbic seizures and status epilepticus—both well-known complication of Herpes encephalitis (49).

Although histopathologic examination was only described in 58% of the cases included in our review, typical features include infiltration with granulocytes and macrophages, necrosis of small vessels and hemorrhages in a “ring and ball” pattern (9, 12, 50). Circumscribed perivascular demyelination seems to occur in cases with a prolonged course of disease, whereas in patients with a fulminant development of AHLE leading to death within 1–2 days from onset, demyelination is usually not (yet) present (31).

Treatment aims at attenuating the autoimmune process believed to cause AHLE, at avoiding secondary neurological damage due to intracranial hypertension and breakdown of the blood-brain-barrier, and at preventing infectious complications, such as pneumonia due to aspiration, that may further promote systemic inflammation.

## Conclusion

Although our systematic review of the literature revealed a lower mortality than previously reported, acute hemorrhagic leukoencephalitis remains a life-threatening neurologic emergency with high mortality. Diagnosis is challenging as the level of evidence regarding the diagnostic yield of clinical, neuroimaging, and laboratory characteristics remains very low. Hence, clinicians are urged to heighten their awareness and to prompt cerebral biopsies in the context of rapidly progressive neurologic decline of unknown origin with the concurrence of the compiled characteristics. Future studies need to focus on treatment characteristics and their effects on course and outcome.

## Ethics Statement

Written informed consent was obtained from the patient's family for the publication of any potentially identifiable images or data included in this article.

## Author Contributions

PG, MS, and RS planned the work, acquired the data, and wrote the manuscript. GD, KT, SR, SM, and JF interpreted the data, revised the manuscript and substantially contributed to the inaugural draft. All authors approved the final submitted version.

## Conflict of Interest

The authors declare that the research was conducted in the absence of any commercial or financial relationships that could be construed as a potential conflict of interest.
